# Supplementary material for: Water Saturated with Pressurized CO2 as a Tool to Create Various 3D Morphologies of Composites Based on Chitosan and Copper Nanoparticles
Source: Molecules. 2022 Oct 26;27(21):7261. doi: 10.3390/molecules27217261 (PMC9658215; doi:10.3390/molecules27217261)
Supplement: Supplementary file 1 [file molecules-27-07261-s001.zip › molecules-1952828-supplementary.pdf]

Supplementary Materials

Water Saturated with Pressurized CO_2 as a Tool to Create Various 3D Morphologies of Composites Based on Chitosan and Copper Nanoparticles

Table of contents

FIGURE S1. Rheological studies.

S1. Rheological behavior discussion.

TABLE S1. XPS studies.

TABLE S2. FTIR spectroscopy data.

FIGURE S2. Photo and SEM of highly porous Cu/CS cryogels.

TABLE S3. Elemental analysis of Cu/CS spherical composites.

FIGURE S3. Photo of Cu/CS spherical composites in different pH media .

TABLE S4. XPS quantification data determined from the survey spectra.

FIGURE S4. O 1s and Cu 2p X-ray photoelectron spectra.

TABLE S5. Amounts of Cu absorbed in Cu/CS sphere.

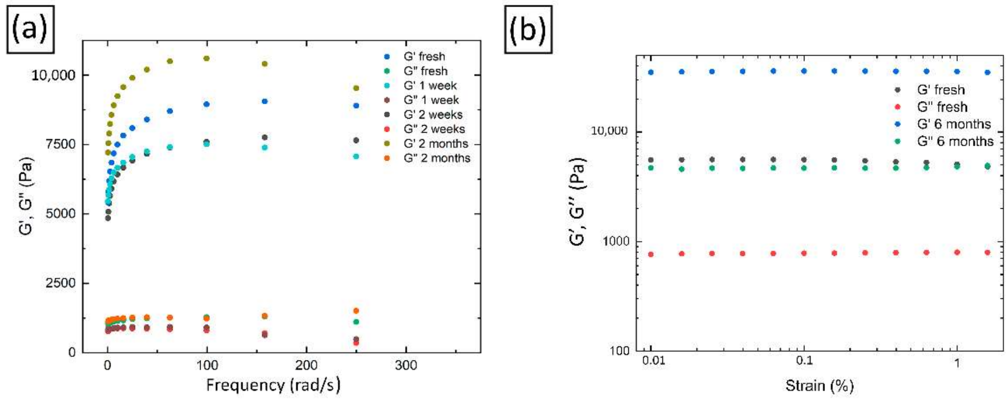

Figure S1. (**a**) Kinetics of rheological properties of Cu/CS composites with different exposure time (low molecular weight chitosan, molar ratio Cu/CS=1/5); (**b**) Strain sweep tests of Cu/CS composites with different exposure time.

S1. Rheological Behavior Discussion

Before the discussion, it should be noted that the results presented below are approximate, since each measurement was carried out on only one sample.

As it can be seen from the graphs in Figure S1 a, G' for the one week gel is less than the G' value for the freshly prepared composite. This can be explained by the presence of rather large clusters of Cu nanoparticles in the one week sample (see more detailed discussion in section 2.2 of the article), that are more difficult to be retained in the network structure by coordination interactions, due to which the gel becomes less elastic. Such an effect was explained by Askadskii et al. [1]. As it is known, crosslinked polymer networks consist of crosslinked points and linear sections between them. However, the description

of the rheological characteristics of coordination networks cannot be the same as for chemically crosslinked gels, since the theory for them assumes that the van der Waals volume of the crosslink node is several times smaller than the van der Waals volume of the linear section of the chain between the crosslinks. In the case of a coordination interactions, such crosslinks are ions, or nanoparticles, or clusters, therefore, the total van der Waals volume of the nodes is approximately equal to the volume of the linear regions between the crosslinks, or can be even larger. This circumstance leads to the fact that when describing the elastic properties of a polymer network, one cannot neglect the ratio of the total van der Waals volume of the crosslinking sites to the volume of linear fragments. Askadskii and co-authors proposed a formula for elastic modulus of crosslinked polymer networks [2]:

$$E = \frac{3\rho R}{Mc}\left(1 + \frac{M_0}{Mc}\beta\right), \quad (1)$$

where ρ is the density, R is the universal gas constant, T is the temperature, M_0 is the molecular weight of the repeating unit in the internodal fragment, M_c is the molecular weight of the internodal fragment, and β defines as

$$\beta = \frac{(\sum_i \Delta Vi)y}{(\sum_i \Delta Vi)0}, \quad (2)$$

where $(\sum_i \Delta V_i)_y$ and $(\sum_i \Delta V_i)_0$ are van der Waals volumes of the network crosslinked point and linear fragment, respectively. The coefficient β was calculated elsewhere [3]; according to the calculation, as the size of nanoparticles increases, the modulus of elasticity decreases. Since, taking into account the relations between G and E, in our case, the size of clusterites is several times greater than the size of individual copper nanoparticles, a deterioration in the rheological properties of the gel can be observed. Is should be noted that after some time the rheological properties of the hydrogel are restored, which is explained by the destruction of the aforementioned clusters.

In the Figure S1 b one can see the region of linear viscoelasticity, where the storage modulus is more than 5 times larger than the loss modulus for both hydrogel samples with different exposure time (at least in the region of deformation less than 1.58%), i.e. the elastic response of the system prevails over the viscous, which is typical for soft solid behavior of physical chitosan gel. Moreover, this tendency persists over the time.

Table S1. Characteristics of the photoelectron spectra: binding energies (E_b), Gaussian widths (W), and relative intensities (I_{rel}) of photoelectron peaks belonging to different chemical groups in the C 1s and N 1s spectra.

**Sample**		**C 1s**								**N 1s**			
	**Group**	**C-C/C-H**	**CH_3-C(O)**	**C-N**	**C-OH**	**O-C-O**	**C(O)N**	**C(O)O**	**CO_3,**	**NH_2**	**N(O)C**	**NH_3^+**	**NO_3, ONO_2**
	**Peak**	**C1**	**C2**	**C3**	**C4**	**C5**	**C6**	**C7**	**C8**	**N1**	**N2**	**N3**	**N4**
CS	E_b (eV)	284.93	285.18	285.72	286.73	288.16	288.68	-	289.44	399.57	400.64	402.16	406.85
	W (eV)	0.88	0.88	1.07	1.06	1	1	-	1.1	1.15	1.15	1.15	1.24
	I_{rel}	0.11	0.02	0.14	0.55	0.14	0.02	-	0.02	0.82	0.13	0.04	0.01
Cu/CS	E_b (eV)	284.96	285.22	285.75	286.73	288.14	288.72	289.72	-	399.58	400.33	401.93	-
	W (eV)	0.91	1.00	1.00	1.02	0.96	1.00	1.00	-	1.22	1.17	1.22	-
	I_{rel}	0.21	0.03	0.12	0.48	0.12	0.03	0.01	-	0.34	0.52	0.14	-
	E_b (eV)	284.8	-	-	286.4	-	-	288.9	-	-	-	-	-

Table S2. FTIR-spectral characteristics of the initial chitosan and Cu/CS composites (low molecular weight chitosan, molar ratio Cu/CS=1/10).

	**Pure CS**		**Cu/CS 1 week**		**Cu/CS 6 weeks**	
	**Wavenumber (cm^{-1})**	**Relative intensities**	**Wavenumber (cm^{-1})**	**Relative intensities**	**Wavenumber (cm^{-1})**	**Relative intensities**
Overlapping stretching of hydrogen bounded –OH and –NH_2	3445	6.7	3291	2.4	3358	3.0
N-H stretching vibration in NH_2	1589	0.8	1569	1.0	1566	1.0
–CH symmetric bending vibrations in -CHOH	1378	1	1379	1	1379	1
C–OH stretching vibrations	1095	6.7	1099	1.8	1093	1.6

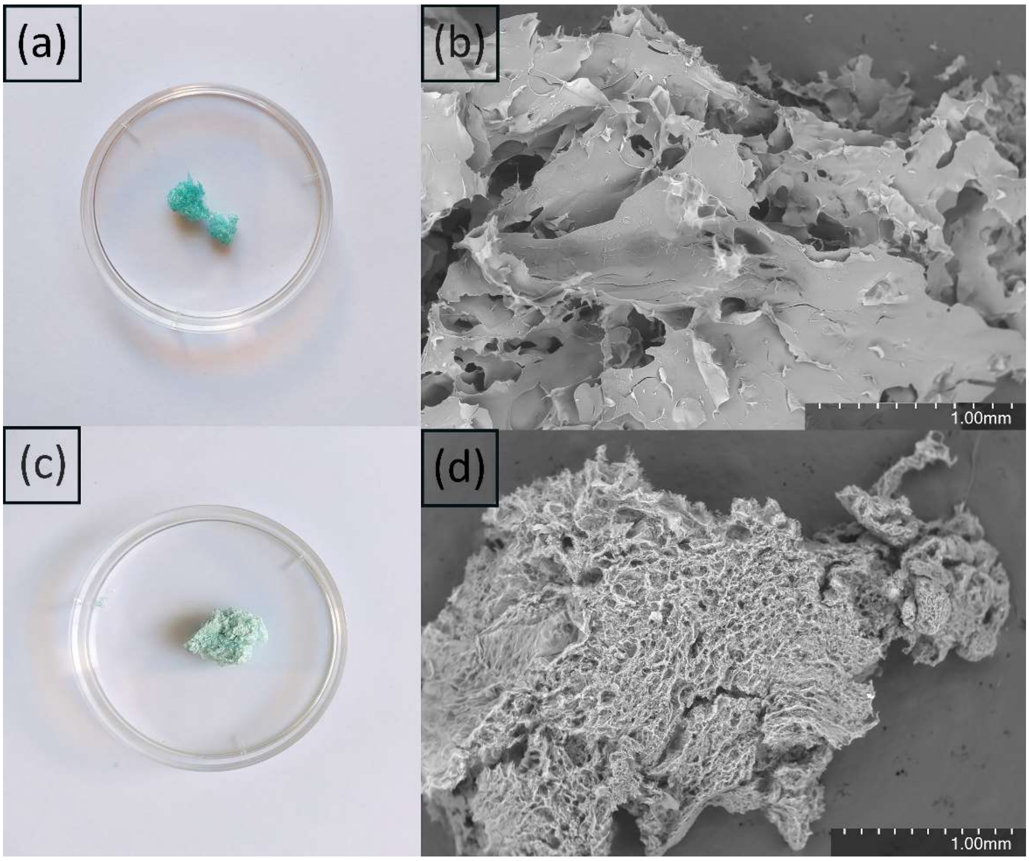

Figure S2. Photographs Cu/CS gel samples (low molecular weight chitosan, molar ratio Cu/CS=1/5) (**a**) saturated with CO_2 (30 MPa); (**c**) modified H_2 (1 MPa) and CO_2 (30 MPa); (**b**), (**d**) – SEM-microphotographs of samples (a) and (c), respectively.

Table S3. Elemental analysis of Cu/CS spherical composites (high molecular weight chitosan, 0.116 m, 300 μL) data.

	**Cu**	**O**
Internal layer (%)	9.9±0.1	31.3±0.2
Transition layer (%)	15.0±0.1	32.3±0.2
Surface layer (%)	26.5±0.2	6.3±0.1

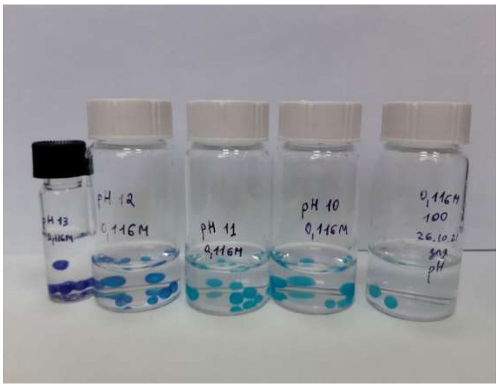

Figure S3. Cu/CS spherical composites (high molecular weight chitosan, 0.116 M, 100 μL) in different pH media**.**

Table S4. XPS quantification data (at. %) determined from the survey spectra.

Sample	O	C	N	Cu	S	Na
Cu/CS	25.3	67.4	4.7	1.0	0.9	-
Cu/CS pH 13	23.7	68.9	4.0	1.1	-	1.5

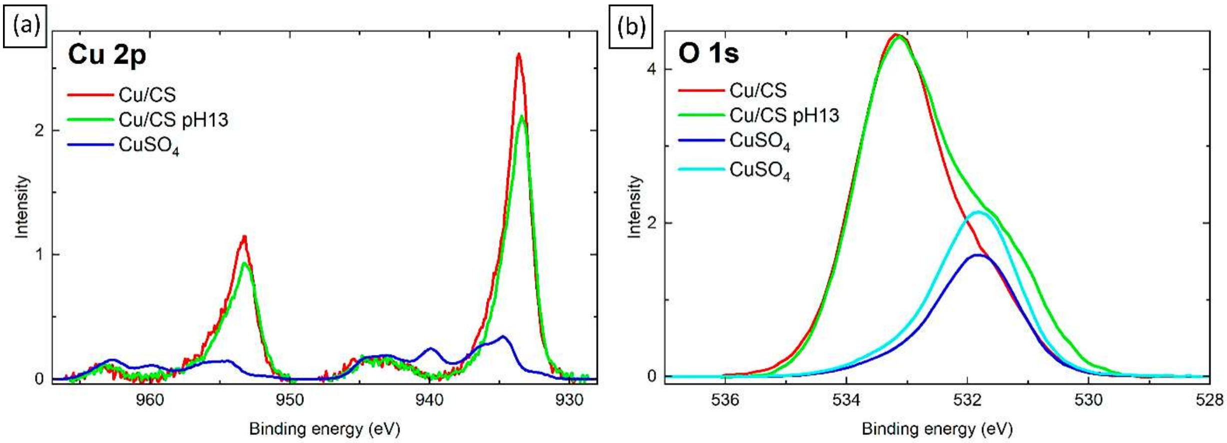

Figure S4. O 1s (**a**) and Cu 2p (**b**) X-ray photoelectron spectra for the initial spherical Cu/CS composite (high molecular weight chitosan, 0.116 M, 100 μL) and the same sample after exposure to the solution with pH 13.

Table S5. Masses of Cu/CS freeze-dried spheres with a different volume of chitosan added and amounts of Cu absorbed in each one.

Chitosan volume (μm)	Sample mass (mg)	Cu mass (mg)
50	0.70 ± 0.07	0.052 ± 0.005
100	1.32 ± 0.11	0.141 ± 0.012
200	2.39 ± 0.10	0.299 ± 0.013

References

1. Askadskii, A. A.; Matveev Yu. I.; Matveeva T. P. The generalized equation for evaluation of the equilibrium modulus of elasticity and Mc value for thin and dense networks. *Polym. Sci. Ser. A* **1988**, 12 2542-2550.
2. Askadskii, A. A. Methods for Calculating the Physical Properties of Polymers. *Rev. J. Chem.* **2015**, 5 (2), 83–142.
3. Matseevich, T.A.; Kovriga, O.V.; Askadskii, A.A. Theoretical analysis of the influence of chemical composition and mixing ration of polymer-solvent nanoparticles on the glass transition temperature. *Plast. massy* **2016,** 7-8, 48-52. (In Russ.)
